# Protein aggregation activates erratic stress response in dietary restricted yeast cells

**DOI:** 10.1038/srep33433

**Published:** 2016-09-16

**Authors:** Ankan Kumar Bhadra, Eshita Das, Ipsita Roy

**Affiliations:** 1 Department of Biotechnology, National Institute of Pharmaceutical Education and Research (NIPER), Sector 67, S.A.S. Nagar, Punjab 160 062, India

## Abstract

Chronic stress and prolonged activation of defence pathways have deleterious consequences for the cell. Dietary restriction is believed to be beneficial as it induces the cellular stress response machinery. We report here that although the phenomenon is beneficial in a wild-type cell, dietary restriction leads to an inconsistent response in a cell that is already under proteotoxicity-induced stress. Using a yeast model of Huntington’s disease, we show that contrary to expectation, aggregation of mutant huntingtin is exacerbated and activation of the unfolded protein response pathway is dampened under dietary restriction. Global proteomic analysis shows that when exposed to a single stress, either protein aggregation or dietary restriction, the expression of foldases like peptidyl-prolyl isomerase, is strongly upregulated. However, under combinatorial stress, this lead is lost, which results in enhanced protein aggregation and reduced cell survival. Successful designing of aggregation-targeted therapeutics will need to take additional stressors into account.

Huntington’s disease (HD) is associated with the aggregation of mutant huntingtin, which harbours an elongated polyglutamine stretch at its N-terminus^1^. The exact role of the native and non-native monomers, oligomers, protofibrils, mature fibrils, etc., in disease pathogenesis remains ambiguous^2^,^3^,^4^. However, the level of aggregation has been positively correlated with disease progression^5^. HD is characterized by the failure of the cellular proteostasis network (PN), comprising of protein synthesis, folding, stress response, degradation and trafficking, to deal with the consequences of protein misfolding and aggregation, which is a hallmark of this and other diseases of this family^6^,^7^,^8^,^9^,^10^,^11^. Ageing has commonly been linked to the deleterious consequences observed in many of these progressively neurodegenerative disorders. A mechanistic link has been proposed between ageing, aggregation-induced proteotoxicity and proteostasis collapse^12^. For example, chaperone genes induced during ageing overlapped with the genes induced during HD. Comparative analysis of gene expression in young and aged human brains showed downregulation of the ‘chaperome’, including TRiC, with age^12^. Interaction of TRiC with mutant huntingtin is important in maintaining the latter in the soluble form^13^,^14^,^15^. Reduced expression of this chaperonin, along with others, during ageing is likely to aggravate the adverse consequences of proteotoxicity. Continued expression of metastable and aggregation-prone proteins reduces the protein folding ability of the cell^16^ implying that chronic stress does not lead to a continuous adaptive response. Signalling by the Ire1 (inositol-requiring enzyme 1) and Atf6 (activating transcription factor 6) branches of the unfolded protein response (UPR) pathway is attenuated upon prolonged endoplasmic reticulum (ER) stress in human cells^17^. Unmitigated ER stress promotes apoptosis^18^,^19^. Prolonged activation of heat shock factor 1 (Hsf1) is linked to improper folding and trafficking defects in CFTR seen in cystic fibrosis^11^. Thus, gene expression changes induced by ageing (one stress) can modulate the cellular response by PN during protein aggregation (second stress) and vice versa. That crosstalk occurs between various pathways is known. The metabolic factor Sir2 acts as a link between the heat shock and UPR pathways^20^. Coupling of UPR to autophagy has been observed in HD model organisms^21^. However, studies carrying out a systematic analysis delineating the effect of each stress response pathway on the overall response of the cell is not widely reported.

Dietary restriction (DR) refers to a dietary regime low in calories without under nutrition. It slows down ageing in a variety of organisms^22^,^23^,^24^. It also reportedly reduces age associated neuronal loss in mouse models of Alzheimer’s^25^ and Parkinson’s^26^ diseases. The low intensity stress induced by DR has been shown to increase life span in yeast^27^. Due to ethical and methodologic considerations, most of these studies are carried out in flies, worms and mostly yeast^22^,^28^,^29^. The cellular mechanism of proteostasis maintenance declines with ageing^30^ and with the onset of protein misfolding disorders^6^,^7^,^8^,^10^. In this work, we have investigated the effect of two simultaneously acting stress factors on the response of the stress response pathway. Specifically, we have studied the effect of DR on a disease caused due to proteotoxicity in the well-validated yeast model of HD.

## Results

### Dietary restriction increases aggregation of mutant huntingtin in yeast cells

**Figure 1:**
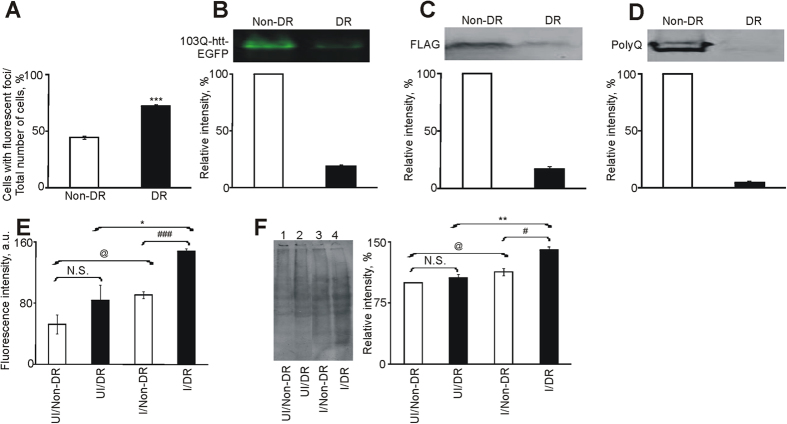
Solubility of 103Q-htt is reduced in yeast cells exposed to dietary restriction. (**A**) Quantification of cells expressing 103Q-htt aggregates was carried out by counting the total number of cells and those exhibiting fluorescent foci in cells grown under non-DR (empty bar) and DR (filled bar) conditions. Cells were counted in 40 different fields. 100% refers to total number of cells counted across 40 fields. ***p < 0.001 against non-DR cells. (**B**) Native PAGE analysis of soluble fraction of yeast cell lysates was carried out. Densitometry of bands was performed using ImageQuantTL (GE Healthcare). Immunoblotting of soluble fraction of cell lysates using (**C**) FLAG and (**D**) polyglutamine (polyQ) antibodies was performed in yeast cells expressing 103Q-htt under non-DR (empty bar) and DR (filled bar) conditions. Intensity of band for 103Q-htt expressed in yeast cells grown under non-DR condition was assigned an arbitrary value of 100% in each of the above cases (**B–D**). (**E**) Measurement of oxidative stress in yeast cells. N.S. = Non-significant; ^@^p < 0.05 against uninduced yeast cells under non-DR condition; ^###^p < 0.001 against induced yeast cells under non-DR condition; *p < 0.05 against uninduced yeast cells under DR condition. (**F**) Estimation of oxidative damage to proteome using DNP (dinitrophenol) antibody. Lane 1: Uninduced cells (non-DR); Lane 2: Uninduced cells (DR); Lane 3: Induced cells (non-DR); Lane 4: Induced cells (DR). Bar graph shows densitometric analysis of the intensities of bands (complete lanes) with Image QuantTL (GE Healthcare). ^@^p < 0.05 against uninduced (UI) yeast cells under non-DR condition; ^#^p < 0.05 against induced (I) (expressing 103Q-htt) yeast cells under non-DR condition; **p < 0.01 against uninduced yeast cells under DR condition. The intensity of the lane for uninduced cells (non-DR) was assigned an arbitrary value of 100%. For panels B–D, cropped gels/blots are shown. All gels/blots have been run under the same experimental conditions. Values shown are mean ± s.e.m. of three independent experiments. Empty bar: non-DR; Filled bar: DR.

N-terminal fragment of human mutant huntingtin (103Q-htt) was expressed as a fusion protein with N-terminal FLAG and C-terminal EGFP tags in *Saccharomyces cerevisiae* BY4742 cells^31^. Fluorescence microscopy revealed the presence of characteristic pin-pointed green fluorescent foci in cells grown under normal (2% dextrose, non-DR) condition, indicating the formation of aggregates by 103Q-htt (Fig. S1A). These fluorescent foci were formed at an earlier time point (3 h) when cells were grown under dietary restricted (0.2% dextrose, DR) condition (Fig. S1A) as compared to non-DR condition (7 h), indicating that DR accelerated aggregation of 103Q-htt. The latter showed the presence of higher number of cells with aggregates of 103Q-htt (72.2 ± 1.7%) as compared to non-DR cells (44.7 ± 1.3%) (p < 0.0002) (Fig. 1A), confirming higher aggregation of 103Q-htt when cells were exposed to DR. Native PAGE analysis showed significantly lower intensity of the band for soluble 103Q-htt in DR cells (five-fold) than in non-DR cells (Figs 1B, S1B), indicating that a higher amount of 103Q-htt was partitioned off into the insoluble fraction in DR cells. Reduced solubilisation of 103Q-htt in DR cells was also confirmed by immunoblotting with FLAG (Figs 1C, S1C) and polyQ (Figs 1D, S1D) antibodies. Thus, yeast cells exhibited earlier and higher aggregation of 103Q-htt upon dietary restriction.

As DR cells were grown at a lower concentration (0.2%) of dextrose prior to induction, it is possible that when transferred to galactose-containing medium, yeast cells metabolize this sugar rather than use it as an inducer, which may interfere with the observations. Yeast cells subjected to DR did not show any difference in growth pattern when compared with non-DR cells (Fig. S1E), indicating that galactose acted only as an inducer of 103Q-htt expression in both cases. The effect of a higher concentration of dextrose on repression of *Gal1* promoter and hence delayed expression and aggregation of 103Q-htt was investigated in non-DR cells. For this, 103Q-htt was expressed downstream of *Cup1* promoter (using the low copy number plasmid pRS315-*CUP1-FLAG-N*_*17*_*-103Q-mRFP* which expresses 103Q-htt at a lower level and hence results in lower aggregation^32^) which responds to Cu^2+^ ions and is not repressible by dextrose. Similar pattern of reduced aggregation of 103Q-htt was observed in non-DR and DR cells (Fig. S1F) as with galactose as an inducer, confirming that aggregation of 103Q-htt is accelerated under DR.

### Enhanced aggregation of 103Q-htt in response to DR correlates with various deleterious phenotypes

The presence of the elongated polyQ stretch and the resultant protein aggregation is linked with a number of deleterious phenotypes. For example, aggregation of mutant huntingtin in HD is associated with higher oxidative stress^33^,^34^. Accordingly, the level of reactive oxygen species (ROS) was found to be higher in both non-DR and DR cells as compared to the corresponding uninduced cells (Fig. 1E). Uninduced yeast cells did not show any significant difference in the levels of ROS under non-DR and DR conditions. Higher level of oxidative stress in DR cells (Fig. 1E) correlated well with higher aggregation of 103Q-htt seen in these cells (Fig. 1A). Higher oxidative stress leads to greater damage to the cellular proteome not only in HD but also in other diseases^33^,^35^. Oxidation of certain amino acid residues leads to the generation of carbonyl groups and has been used as a marker of ROS-induced oxidative damage to proteins^36^. Carbonyl groups were derivatized using dinitrophenylhydrazine and oxidative damage was detected using dinitrophenol (DNP) antibody^37^. Uninduced yeast cells subjected to DR did not show any significant difference in oxidative damage than non-DR cells (Fig. 1F). Yeast cells expressing 103Q-htt under non-DR condition showed marginally higher level of oxidatively modified proteins than the corresponding uninduced cells, while significantly higher proteomic damage was seen in cells expressing 103Q-htt under DR condition than the corresponding uninduced cells (Fig. 1F). Significantly higher damage to the proteome was also seen in DR cells expressing 103Q-htt than in the corresponding non-DR cells (Fig. 1F). These results correlated well with the higher level of aggregation of 103Q-htt (Fig. 1A) and oxidative stress (Fig. 1E) generated in these cells.

**Figure 2:**
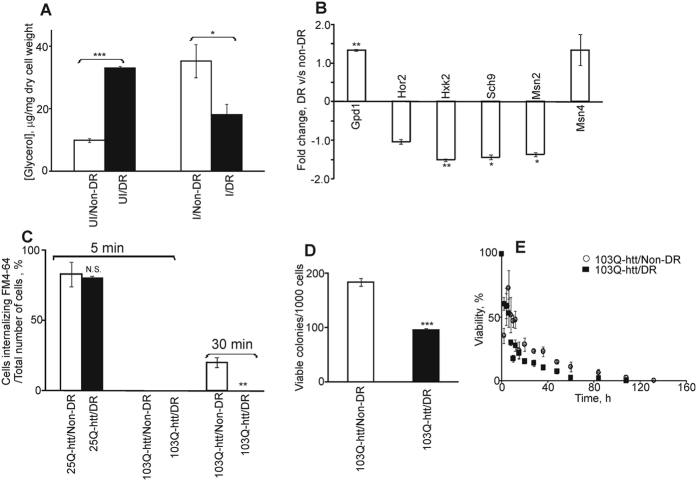
Increased aggregation of 103Q-htt has adverse effect on survival of yeast cells subjected to DR. (**A**) Estimation of level of glycerol in yeast cells. ***p < 0.001, *p < 0.05 against corresponding non-DR condition. (**B**) Comparative analysis of expression of genes involved in HOG pathway in yeast cells expressing 103Q-htt grown under non-DR and DR conditions. Differential expression of genes expressed in cells grown under DR condition was measured against that under non-DR condition. **p < 0.01, *p < 0.05 against cells grown under non-DR condition. (**C**) Quantification of endocytosis in yeast cells expressing 25Q-htt or 103Q-htt. Comparison of total number of cells with internalization of FM4-64 under non-DR and DR conditions. 100% refers to internalization of FM4-64 by all cells exhibiting fluorescence of EGFP (due to 25Q-htt or 103Q-htt). (**D**) Viability of yeast cells expressing 103Q-htt was measured by counting the number of colonies seen on SC-URA + 2% dextrose plates. ***p < 0.001 against non-DR condition. (**E**) Measurement of chronological life span of yeast cells expressing 103Q-htt. 100% represents viability of starting cells in each case. Values shown are mean ± s.e.m. of three independent experiments. For panels A,C,D: Empty bar: non-DR; Filled bar: DR.

DR leads to the synthesis of glycerol in yeast^38^. Intracellular glycerol is involved in osmo-adaptation, oxidative stress protection and response to heat shock^39^. In agreement with this, uninduced yeast cells subjected to DR showed more than three-fold increase in glycerol production than non-DR cells (Fig. 2A). Increased glycerol level during DR is attributed to the compensatory mechanism which becomes operational due to increased glycolytic flux and compromised cellular respiration^38^. Upon expression of 103Q-htt, non-DR cells showed more than three-fold increase in the level of intracellular glycerol compared to uninduced cells. Thus, when exposed singly to both types of stress conditions, either DR or aggregation of 103Q-htt, the level of glycerol increased. Upon expression of 103Q-htt, DR cells showed more than two-fold reduction in the level of glycerol as compared to uninduced DR cells (Fig. 2A). Thus, a combination of stress conditions does not activate the osmoregulatory pathway of glycerol production to the same level as individual stress conditions do. The transcript level of the genes involved in the high osmolarity glycerol (HOG) pathway^40^ was measured by reverse transcription real-time polymerase chain reaction. Except for *Gpd1* (glycerol 3-phosphate dehydrogenase 1), expression of all other genes, like *Hor2* (glycerol 3-phosphate phosphatase), *Hxk2* (hexokinase), *Sch9* (orthologue of mammalian S6K and Akt/PKB) and *Msn2* (stress-responsive transcriptional activator) was significantly downregulated in cells expressing 103Q-htt upon DR (Fig. 2B). Long-lived mutants (Δ*Sch9*) have been reported to show induction of glycerol biosynthetic genes^38^. Addition of glycerol to the culture medium in this case did not stimulate pro-ageing signalling events in the same manner as addition of dextrose of ethanol^38^. Reduced level of glycerol observed in DR cells, despite downregulation of *Sch9*, hints at the role of aggregation of 103Q-htt in modulating intracellular osmoregulation. The effect of addition of glycerol to this dual stressed cells remains to be seen.

Normal huntingtin (<36Q) is involved in protein trafficking in cells^41^. Elongation of polyQ tract at the N-terminus (resulting in mutant huntingtin) shows disruption of endocytosis^42^. Endocytosis was monitored using the lipophilic dye, N-[3-triethylammoniumpropyl]-4-[p-diethylaminophenylhexatrienyl] pyridinium dibromide (FM4-64)^42^. Cells expressing 25Q-htt-EGFP (non-pathogenic) demonstrated a labelling pattern characteristic of normal endocytosis; red patched structures, representing labelled endosomes, were seen in the cytoplasm within the first 5 min of addition of the dye (Fig. S2). This was independent of whether the cell was grown under non-DR or DR condition. Cells expressing 25Q-htt under non-DR (83 ± 8.73%) and DR (80 ± 1.45%, p < 0.7783) conditions did not show any significant difference in internalization of FM4-64 after 5 min (Fig. 2C). Thus, DR alone did not have any effect on endocytosis in yeast cells. In contrast, almost none of the cells (either non-DR or DR) expressing 103Q-htt contained labelled endosomes or fluorescent rings surrounding vacuoles after 5 min (Fig. S2), indicating that aggregation of 103Q-htt delayed endocytosis^42^. Non-DR cells expressing 103Q-htt showed internalization of FM4-64 after 30 min, while internalization was not seen in DR cells expressing 103Q-htt even after 30 min (Fig. S2). A significantly higher number of cells expressing 103Q-htt (20 ± 3.52% vs. none) showed internalization of FM4-64 after 30 min under non-DR as compared to DR condition (Fig. 2C). These results suggested that the impairment of endocytosis was stronger in DR cells showing higher aggregation of 103Q-htt.

An imbalance in the rates of oxidation of proteins and their degradation leads to a build-up of toxic intermediates with adverse effect on cell survival. Viability of DR cells expressing 103Q-htt was found to be significantly lower than non-DR cells (Fig. 2D), correlating well with the increased level of aggregated protein and oxidative damage to the proteome in the former case. The ability of yeast to resume mitotic growth when transferred to a fresh medium after they have entered a non-dividing state upon exhaustion of carbon source is measured as chronological lifespan (CLS)^43^,^44^. DR is reported to extend CLS in yeast and longevity in some other organisms^45^,^46^. Uninduced DR cells showed significantly higher longevity (30 days) as compared to non-DR cells (12 days) (Fig. S3). However, longevity was found to be higher in induced non-DR (t_1/2_ 5.5 days) as compared to DR (t_1/2_ 4.5 days) cells (Fig. 2E), matching the higher aggregation of 103Q-htt (Fig. 1A) in DR cells. Thus, yeast cells expressing 103Q-htt under DR showed enhanced aggregation of protein correlating with reduced glycerol, increased oxidative stress, impaired endocytosis, and decreased cell viability and longevity. On the other hand, DR had no effect on the growth of cells expressing 103Q-htt (Fig. S1B). This comparison leads to the speculation that DR confers a higher ‘fitness advantage’ on the surviving cells and may be correlated with the viewpoint that formation of mature aggregates is a cytoprotective event^3^.

### Activation of stress response pathways is not a cumulative effect

**Figure 3:**
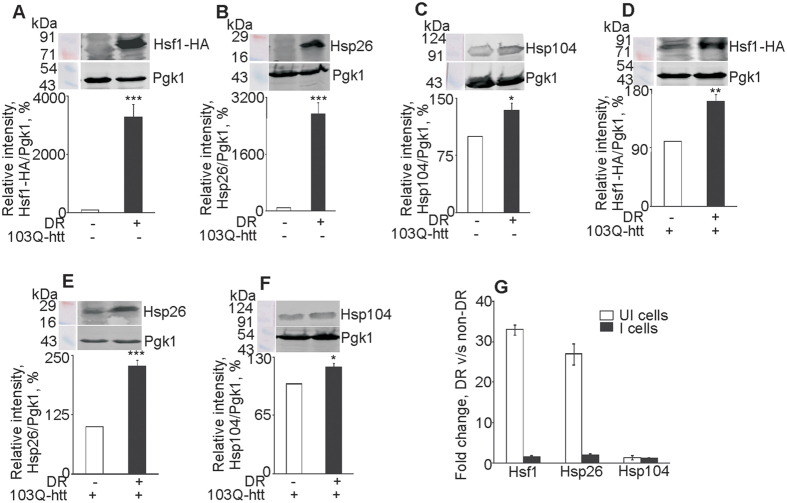
Heat shock response in yeast cells. Immunoblotting of (**A–C**) uninduced and (**D–F**) induced yeast cells grown under non-DR and DR conditions was carried out with (**A,D**) HA (for Hsf1-HA), (**B,E**) Hsp26 and (**C,F**) Hsp104 antibodies. The ratio of intensity of band for the desired protein to the band for loading control (Pgk1) in (**A–C**) uninduced or (**D–F**) induced cells under non-DR condition was assigned an arbitrary value of 100% in each case. *p < 0.05, **p < 0.01, ***p < 0.001 against corresponding cells grown under non-DR conditions. (**G**) Comparison of expression levels of Hsf1, Hsp26 and Hsp104 in uninduced (UI) and induced (I) cells under non-DR and DR conditions. For panels A–F, cropped blots are shown. All blots have been run under the same experimental conditions. Values shown are mean ± s.e.m. of three independent experiments.

The level of glycerol increased in response to aggregation of 103Q-htt and DR, when monitored individually, but showed a reverse trend when expression of 103Q-htt was monitored in DR cells (Fig. 2A). The level of response did not correlate with the magnitude of stress, especially after two stress conditions were combined. Hence, we decided to monitor the level of some proteins involved in stress response pathways in yeast. Heat shock response (HSR) in yeast cells is mediated by Hsf1 through the downstream activation of a number of heat shock proteins, including Hsp104 and Hsp26. The expression of Hsf1 was found to be thirty three-fold higher in uninduced yeast cells under DR as compared to the corresponding non-DR cells (Fig. 3A). Similarly, the expression of Hsp26 was found to be twenty seven-fold higher in uninduced DR cells (Fig. 3B). The expression of Hsp104 was also marginally but significantly higher in uninduced DR cells (Fig. 3C). Thus, HSR is activated in yeast cells in response to DR. Activation of Hsf1 in response to calorie restriction in yeast has been reported earlier^47^. DR and HSR have a synergistic effect on expression of Hsp70 in *Caenorhabditis elegans*^48^. Deacetylation of Hsf1 by the metabolic factor SIRT1 modulates longevity in HeLa cells by prolonging the binding of Hsf1 to the heat shock promoter Hsp70^49^. This provides another clue to the coupling of dietary regimen to HSR and proteostasis. We and others have shown that HSR is not activated in yeast cells upon aggregation of mutant huntingtin^50^,^51^. The expression of Hsf1 was found to be only 1.5-fold higher in DR cells expressing 103Q-htt as compared with the same cells under non-DR condition (Fig. 3D). This was interesting as the expression of Hsf1 was strongly upregulated in DR cells in the absence of 103Q-htt expression. Since HSR is not induced by proteotoxic stress in yeast, one would have expected that the expression of Hsf1 would remain high in DR cells with aggregation of 103Q-htt (i.e. remain the same as in uninduced DR cells), which was not the case. A similar mild increase in expression was seen for Hsp26 (Fig. 3E) and Hsp104 (Fig. 3F). The strong overexpression of Hsp26 seen with DR cells not expressing 103Q-htt was dampened when expression of the mutant protein was induced (Fig. 3G). Since Hsp26 is a client protein of Hsf1, a second possible reason could be that the reduced expression of Hsf1 did not allow the expression of Hsp26 to go up. Another plausible explanation for the attenuated response is that the surviving cells under DR conditions have an increased replicative capacity which confers on them an increased survival advantage, as has been discussed above. This can also be correlated with the observation that formation of inclusion bodies is beneficial for the affected cell^3^. The results confirmed the attenuation of HSR with a combination of stressors (DR and aggregation of 103Q-htt) (Fig. 3G) and indicated that the cell does not respond to a multitude of stress conditions by activating an infinite response. This was further confirmed by transcriptome analysis of some members of the heat shock protein family (Fig. S4). The expression of a majority of genes, viz. *Hsf1, Hsp104, Hsp70*s (*Ssa2* and *Ssa3*) and Type I Hsp40 (*Ydj1*) was downregulated when cells expressed 103Q-htt under DR condition as compared to induced cells grown under non-DR condition.

**Figure 4:**
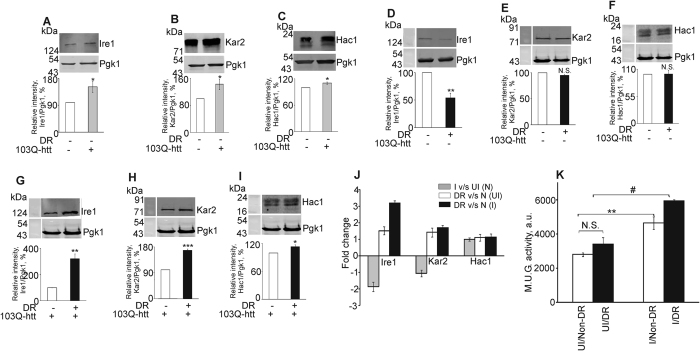
Induction of unfolded protein response in yeast cells. Immunoblotting of (**A–C**) uninduced (UI) and induced (I) yeast cells grown under non-DR conditions, (**D–F**) uninduced and (**G–I**) induced yeast cells grown under non-DR and DR conditions was carried out with (**A,D,G**) Ire1, (**B,E,H**) Kar2 and (**C,F,I**) Hac1 antibodies. The ratio of intensity of band for the desired protein to the band for loading control (Pgk1) was assigned an arbitrary value of 100% in each case. N.S. Non-significant, *p < 0.05, **p < 0.01, ***p < 0.001 against corresponding uninduced cells (**A–C**) or cells grown under non-DR conditions (**D–I**). (**J**) Comparison of results for experiments performed under different conditions. (**K**) Unfolded protein response was measured using the *UPRE-lacZ* reporter construct. Yeast cells were cotransformed with pYES2-*103Q-htt* and *UPRE-lacZ* reporter construct. Enzyme activity was expressed as M.U.G. (methylumbelliferyl galactopyranose) units. **p < 0.01 against uninduced (UI) non-DR cells, ^#^p < 0.05 against induced (**I**) non-DR cells. For panels A–I, cropped gels/blots are shown. All gels/blots have been run under the same experimental conditions. Values shown are mean ± s.e.m. of three independent experiments.

Stress factors which disrupt the function of the ER lead to accumulation of unfolded proteins in the ER and result in ER stress. Chronic ER stress is implicated in many human diseases^8^,^10^,^52^. Cells react to ER stress by activating an integrated signal transduction pathway called the UPR and restore ER homeostasis^8^,^50^,^53^,^54^,^55^. In *Saccharomyces cerevisiae*, UPR is regulated by the ER stress sensor, Ire1^56^,^57^. To determine whether UPR was activated by the aggregation of 103Q-htt or DR, the expression levels of Ire1 and its clients, Kar2 and Hac1, were monitored. The expression levels of Ire1 (Fig. 4A), Kar2 (Fig. 4B) and Hac1 (Fig. 4C) were significantly upregulated in yeast cells expressing 103Q-htt as compared to uninduced cells under non-DR condition. Thus, misfolding and aggregation of the mutant protein led to activation of UPR. The expression of Ire1 was found to be marginally but significantly downregulated in uninduced DR cells compared with the corresponding non-DR cells (Fig. 4D). However, no difference in the expression levels of Kar2 (Fig. 4E) and Hac1 (Fig. 4F) was observed. The activation of UPR was not that significant when cells were subjected to DR. The expression level of Ire1 increased more than three-fold in DR cells expressing 103Q-htt as compared to corresponding non-DR cells (Fig. 4G). Activation of Ire1 increases aggregation of mutant huntingtin in neuronal cells with ER-stress insult, via the inhibition of autophagy flux^58^. Thus, increased expression of Ire1 in DR cells expressing 103Q-htt correlated well with the enhanced aggregation (Fig. 1A) observed in these cells. Expression levels of Kar2 (Fig. 4H) and Hac1 (Fig. 4I) were also significantly upregulated in DR cells expressing 103Q-htt. The results are summarized in Fig. 4J. The discordant nature of gene expression was obvious when the expression levels of a few UPR target genes were monitored (Fig. S5). The expression of *Lhs1* (coding for a nucleotide exchange factor associated with the lumenal Hsp70, Kar2, localized to the ER) is regulated by the UPR pathway^59^. Lhs1 is predicted to act as a nucleotide exchange factor for Kar2^60^ and its deficiency adversely affects protein folding at the ER lumen^61^. Similarly, the expression of *Ino1* (coding for inositol-3-phosphate synthase) and *Pdi1* (coding for the protein disulphide isomerase responsible for redox homeostasis at the ER) is also strongly induced upon ER stress^55^,^59^. Along with the lectin-like protein Htm1, Pdi1 is involved in the degradation of misfolded glycoproteins^62^. Comparison of DR cells with non-DR cells showed the same pattern of gene expression of the target genes as the expression pattern of Ire1 and other proteins. Similarly, expression of all genes followed the same pattern as expression of Ire1 upon dietary restriction except that of *Pdi1*. Combination of stress conditions led to no significant change in expression of UPR target genes confirming that the expression levels of all three genes in cells expressing aggregated 103Q-htt under DR condition could not have been predicted from their expression levels under proteotoxic or dietary stress alone (Fig. S5). We also measured the unfolded protein response in the cell using a reporter construct (*UPRE-lacZ*)^63^. UPR downstream genes contain UPR element in their promoters which allows their transcription in response to ER stress. Upon aggregation of 103Q-htt, significant activation of UPR was observed when compared with uninduced non-DR cells (Fig. 4K), correlating well with the protein expression levels (Fig. 4A–C). Activation of UPR was not observed in uninduced DR cells (Fig. 4K), matching with the comparative expression levels of the respective proteins in these cells (Fig. 4D–F). UPR was also significantly upregulated in DR cells expressing aggregated 103Q-htt as compared to the corresponding non-DR cells (Fig. 4K). This increase (1.3-fold) was, however, lower than the upregulation of UPR resulting due to aggregation of 103Q-htt alone (1.6-fold). Thus, similar to the mismatch observed in case of HSR, the activation of UPR too was not cumulative in response to stress.

### Global proteomic analysis shows differential expression of proteins under stress conditions

**Figure 5:**
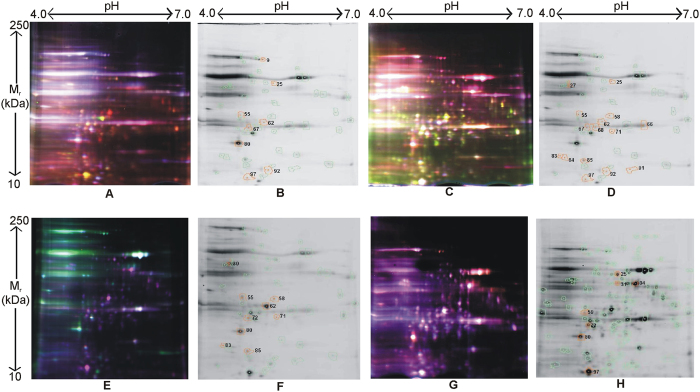
High resolution 2D DIGE proteome analysis of yeast cells under different conditions. Proteins were labelled with Cy dyes. Representative images of gels for (**A,B**) uninduced yeast cells grown under non-DR and DR conditions, (**C,D**) uninduced and induced yeast cells grown under non-DR condition, (**E,F**) uninduced and induced yeast cells grown under DR condition, (**G,H**) induced yeast cells grown under non-DR and DR conditions, are shown, where the fluorescence emission from Cy3 and Cy5 dyes is superimposed (**A,C,E,G**). Orange spots in representative grey-scale images of gels (**B,D,F,H**) indicate differentially expressed proteins which passed the stringent statistical parameters (p < 0.05).

Both DR and aggregation of 103Q-htt are stress conditions for the yeast cell. Activation of stress response machinery is likely to be an adaptive defence mechanism of the cell. Global proteomic analysis was performed using two dimensional difference gel electrophoresis (2D-DIGE) to understand the changes occurring in the cell in response to a combination of stressors. Firstly, the difference in expression of global proteome in uninduced yeast cells was monitored under non-DR and DR conditions (Fig. 5A). In total, 1076 spots were detected, from which 93 spots were selected, each having a suitable 3D landscape. Out of these, 52 spots were selected by matching across gel replicates against a master gel. Eight spots passed the stringent statistical parameters (p < 0.05) (Fig. 5B, surrounded by orange circles). Four of these were significantly upregulated in yeast cells subjected to DR (Table 1). The expression of one protein (spot number 9) was significantly downregulated in DR cells (Table 1). Relative protein abundance for each spot was plotted as log standardized value (Fig. S6).

Next, the difference in expression of global proteome with aggregation of 103Q-htt was monitored (Fig. 5C). A comparative analysis between uninduced and induced non-DR cells was carried out. From a total of 1076 detected spots, 106 spots were selected, having a suitable 3D landscape. Sixty nine spots were selected by matching across gel replicates against a master gel. Fifteen spots passed the stringent statistical parameters (p < 0.05) (Fig. 5D, surrounded by orange circles). Out of 15 spots, five proteins were found to be significantly upregulated in non-DR cells expressing aggregated 103Q-htt as compared to the corresponding uninduced cells (Table 1).

Next, the difference in expression of global proteome in yeast cells showing aggregation of 103Q-htt under DR was monitored (Fig. 5E). A comparative analysis between uninduced and induced DR cells was done. From a total of 1076 detected spots, 101 spots were selected, having a suitable 3D landscape. Nine spots passed the stringent statistical parameters (p < 0.05) (Fig. 5F, surrounded by orange circles). Two proteins were significantly upregulated in DR cells expressing aggregated 103Q-htt as compared to uninduced DR cells (Table 1). The expression of one protein (spot number 62) was significantly downregulated in induced DR cells (Table 1).

Lastly, the differential global proteome expression in yeast cells expressing 103Q-htt under non-DR and DR conditions was monitored (Fig. 5G). Ninety nine spots were selected from a total of 1076 spots. Seven spots passed the stringent statistical parameters (p < 0.05) (Fig. 5H, surrounded by orange circles). Two proteins were identified to be marginally downregulated in yeast cells expressing 103Q-htt under DR as compared to non-DR condition (Table 1).

The results indicated that there were five proteins (spot numbers 25, 58, 62, 71 and 97) which were significantly upregulated in yeast cells upon expression of 103Q-htt. Out of these, three proteins (spot numbers 25, 62 and 97) were also significantly upregulated under DR condition (in the absence of aggregation of 103Q-htt). The expression of two of these three proteins (spot numbers 25 and 97) was marginally downregulated in yeast cells expressing 103Q-htt under DR as compared to non-DR (Table 1). This observation is in line with the earlier results which showed that expression levels of proteins involved in the stress response pathways of the cell do not increase linearly with accumulation of stress. Protein spots of interest were excised, digested with trypsin and analyzed by MALDI-TOF/TOF mass spectrometry (Sandor Life Sciences Pvt. Ltd., Hyderabad, India). A total of seven proteins were identified (Table 2). These were Atp2 (β-subunit of the F_1_ sector of mitochondrial F_O_F_1_ ATP synthase), Eno1/2 (phosphopyruvate hydratase), Arp2 (actin-related protein), Tpi (triose phosphate isomerase), Utp21 (subunit of U3-containing 90S preribosome and SSU processome complex), Hom6 (homoserine dehydrogenase) and Fpr1 (peptidyl-prolyl cis-trans isomerase) (Table 2). Two of these, Eno1/2 and Tpi, are components of the glycolytic cycle while another protein, Atp2, catalyzes the terminal step of oxidative phosphorylation in mitochondria of eukaryotic cells and is directly involved in ATP synthesis. Actin-related proteins (Arp) are highly conserved across species and interact with elongated polyQ stretches in the yeast model of HD via [Rnq1^+^] prion^64^. Utp21 is a component of a multiprotein nucleolar complex involved in ribosome biogenesis. Hom6 catalyzes the conversion of L-aspartate semialdehyde to homoserine which is the third step in the biosynthetic pathways of threonine and serine. The protein whose differential expression level probably best illustrates the effect of individual versus cumulative stressors is peptidyl-prolyl cis-trans isomerase (PPIase, Fpr1, Spot 97). Thus, almost all proteins with altered expression seem to be involved in protein synthesis and maintenance of cellular homeostasis. The identification data for peptide mass fingerprinting and accession numbers are shown in Table S1.

## Discussion

Proteostasis and stress response decline with aging, and are thought to be a major cause of the accumulation of damaged, misfolded and aggregated proteins^9^,^10^. Both HSR and UPR deteriorate in an aging yeast cell^65^. Crosstalk between various arms of PN allows a fine balance to be maintained between synthesis and degradation of proteins, in both ‘normal’ and stress conditions^20^,^21^. Overwhelming the cell with an excess of stress disturbs this equilibrium and misaligns the cellular adaptive response. In this work, we have studied the effect of two stress conditions, DR and proteotoxicity, on the activation of stress response in yeast cells. We did find stress response pathways to be activated to different extents in response to the nature of stress. However, we did not find a cumulative response in either of the two pathways studied, i.e. HSR or UPR. Unmitigated stress led to dampening of the response such that in most cases, the collective response was even lower than individual responses.

In order to find a rationale for this observation, identification of proteins with differential expression under a variety of stress conditions was attempted using 2D-DIGE (Fig. 5). The yeast orthologue of the human FKBP12, cytosolic Fpr1, is one of the few members of the FKBP (FK506-binding proteins) family with an orthologue in higher eukaryotes^66^. FKBP12 is associated with Alzheimer’s disease (AD)^67^ and its expression is reduced in AD patients^68^. This may be because FKBP12 can inhibit the fibrillation of tau due to PPIase activity of the chaperone^69^. Induction of UPR by zinc deficiency resulted in upregulation of FKB2 in *S. cerevisiae*^63^. In yeast, FKBP interacts with Hsf1^70^. Significant upregulation of peptidyl-prolyl isomerase was seen when (uninduced) yeast cells were exposed to DR condition (Fig. 5B) or when mutant huntingtin formed aggregates in yeast cells (under non-DR condition) (Fig. 5D). This correlated well with the above analysis and indicated the cell’s attempts to overcome folding defects. However, when a combination of stress conditions (aggregation of 103Q-htt under DR) was present (Fig. 5H), the expression of PPIase foldase was downregulated and proteostasis could not be restored.

The counter-intuitive results observed in this work are not altogether surprising. DR was unable to attenuate proteotoxicity resulting from aggregation of mutant TDP43 (mimicking) ALS in *C. elegans*^71^ or in Drosophila resulting from aggregation of Aβ^72^. In Tg4510 mice expressing P301L tau, 4 month-long DR regimen was unable to reverse reduction in ADP-stimulated respiratory rates, increased mitochondrial membrane potential and reduced mitochondrial Complex I activity observed in non-transgenic control animals^73^. In MPTP-induced Parkinson’s disease model of rhesus monkeys, all symptoms associated with degeneration of dopaminergic neurons were significantly ameliorated upon DR^74^. Notably, the MPTP model does not lead to aggregation of α-synuclein, which is associated with progression of PD. Thus, the outcome of a therapeutic strategy needs to be validated using suitable disease models as well as different modes and duration of DR. This is obvious from the report that upon DR (40% restriction), the triple transgenic mouse model of AD (3XTgAD) showed reduced levels of Aβ_1–40_, Aβ_1–42_ and phosphorylated tau compared to control mice (fed *ad libitum*) at 17 months age while no such reduction was observed in 3XTgAD mice fed intermittently (food deprivation for 24 h every second day)^75^. A recent report implies that DR has a protective role in full length YAC128 mouse model of HD^76^. The definition of DR (intermittent fasting every second day) and the time of sacrifice (after a day of feeding and not fasting) used in this work seem to influence the results. For example, the transcription levels of a number of metabolic markers remained unchanged^76^, which is surprising. This was also opposite to what the authors had observed earlier when the animals were sacrificed after fasting^77^. Similar to the discrepancy in gene expression observed by us upon a combination of stress conditions as compared to stress in isolation, the expression of certain genes like *Plod2* and *ApoE* was unexpectedly altered upon DR in HD background^76^. DR also shortened lifespan in an ALS (amyotrophic lateral sclerosis) mouse model^78^. Conserved pathways of aging have a synchronised effect on multiple age-related diseases in humans, similar to what has already been shown in model organisms^79^. The presence of additional stress factors or metastable proteins aggravates the toxicity due to polyQ proteins. This has given rise to the “feed-forward” hypothesis of amplification of proteotoxicity^16^. As the protein misfolding disorders progress over a long period of time, outcomes of prolonged activation of stress response pathways are difficult to predict, as these responses are activated due to transient, rather than chronic, proteotoxic stress. In-built within the stress response pathways are mechanisms which downregulate the expression of stress sensors via feedback loops upon prolonged exposure. Failure to activate these ameliorating mechanisms has harmful effects for the cell and the organism. For instance, prolonged activation of HSR either by overexpression of Hsf1 or by addition of an activator molecule aggravated proteotoxicity due to polyQ (91Q) aggregates in U2OS and HEK293 cells^80^. Upon prolonged ER stress in the presence of ER stressors tunicamycin or thapsigargin, signalling by Ire1 and downstream components declined in HEK293 cells^17^. In transgenic rat model of autosomal dominant retinitis pigmentosa caused by misfolding of mutant (P23H) rhodopsin, constitutive expression of the mutant protein initially led to higher transcriptome level of *BiP*, coding for the Hsp70-class ER chaperone, indicating stronger induction of UPR in case of the misfolded protein. Thereafter, *BiP* mRNA level declined, accompanied by increased expression of the proapoptotic transcription regulator *Chop*, which was associated with faster retinal degeneration^17^. Failure of the cell to restore homeostasis during the initial response tenure activates mechanisms which lead to cell death^81^. A possible therapeutic strategy could be development of a titrated approach with specific inhibitors^82^, so that unintended effects can be avoided.

## Methods

### Expression and aggregation of mutant huntingtin

N-terminal fragment of human mutant huntingtin carrying 103 glutamines was expressed as a FLAG- (N-terminal) and EGFP-tagged (C-terminal) protein in *Saccharomyces cerevisiae* cells as described previously^15^,^31^. Transformed yeast cells (pYES2-*FLAG-N*_*17*_*-103Q-EGFP* or pYES2-*FLAG-N*_*17*_*-25Q-EGFP*) were grown in SC-URA medium containing 2% (non-DR) or 0.2% (DR) dextrose and incubated at 30 °C, 200 rpm till A_600_ 0.8. Expression of protein was induced with SC-URA + 2% galactose after centrifuging off the dextrose medium. Fluorescence microscopy (Nikon Eclipse E600, Nikon, Japan) was used to monitor aggregation profile of 103Q-htt under different conditions of growth. For aggregate scoring (Fig. 1A), the number of cells showing characteristic fluorescent foci of aggregated 103Q-htt was counted against the total number of cells in a field. For the coexpression of proteins (Hsf1-HA or UPRE-LacZ), cotransformed cells were grown in SC-URA-LEU medium containing 2% or 0.2% dextrose. We were unable to detect endogenous Hsf1 with anti-Hsf1 antibody^15^. Hence, Hsf1 was coexpressed (along with 103Q-htt-EGFP) as Hsf1-HA in both cells. This could be either due to the inability of the antibody to detect endogenous Hsf1 and/or the convenience of the HA fusion tag as a detection agent^83^. Both proteins were expressed constitutively.

### Analysis of protein expression

At the end of induction period, yeast cells were pelleted down and resuspended in lysis buffer containing TrisHCl (50 mM), NaCl (150 mM), DTT (2 mM), glycerol (10%), Triton X-100 (1%), pH 7.5 supplemented with 1 mM PMSF. Cells were disrupted using acid washed glass beads by vortexing six times for 45 sec with intermittent rests of 30 sec on ice. After lysis, the tubes containing the lysate were set aside for 1 h for the glass beads and cell debris to settle down. The lysate was then transferred to another centrifuge tube and centrifuged at 15,000 g for 50 min. Protein estimation was done in the resulting supernatant by Bradford method using bovine serum albumin as the standard protein. The soluble fractions obtained were analysed by 12% native PAGE. Samples were also resolved by 12% SDS PAGE. The protein bands from denaturing gel were transferred electrophoretically to a nitrocellulose membrane (0.45 μm). After overnight blocking, proteins were visualized using polyQ (1:5,000), FLAG (1:1,000), DNP (1:2,500), HA (1:500), Hsp26 (1:400), Hsp104 (1:1,00,000), Ire1 (1:1,000), Kar2 (1:1,000), Hac1 (1:1,500) and Pgk1 (1:5,000) antibodies. FITC conjugated anti-rabbit (1:1,000) or anti-mouse (1:1,000) antibodies were used as secondary antibodies. The blots were scanned for FITC (λ_ex_ 488 nm; λ_em_ 526 nm) using an image scanner (Typhoon Trio, GE Healthcare).

### Characterization of yeast cells expressing aggregated 103Q-htt

Oxidative stress in cells was measured using 2′,7′-dichlorodihydrofluorescein diacetate (DCFH-DA)^84^. The emission intensity of dichlorofluorescein was recorded using λ_ex_ 504 nm, λ_em_ 519 nm. Cell viability was measured by plating induced cells (1 × 10^3^) on SC-URA + 2% dextrose plates and monitoring growth for 2–3 days at 30 °C. For measurement of CLS, cells were withdrawn at different time points of induction, plated onto SC-URA + 2% dextrose plates and incubated at 30 °C for 2–3 days to get single colonies on plates. The number of single colonies on the plate represented the number of viable cells in the culture. The study was carried out till no colonies were seen on the plate at the lowest dilution^15^,^43^. For monitoring endocytosis, cells were incubated with the lipophilic dye FM4-64 (8 μM) for different time periods^42^. Endocytosis of the dye was monitored using a fluorescence microscope (Model E600, Nikon Corp., Japan; excitation at BP 510–560 nm, emission BA 590 nm). Twenty fields, each containing 40–50 cells, were counted in each case. Cells which exhibited both EGFP and FM4-64 fluorescence were counted as positive cells. Glycerol was estimated using the free glycerol detection kit (Abcam, USA) as per manufacturer’s instructions.

### Gene expression analysis

Total RNA was isolated from yeast cells by the hot phenol method^85^. RNA (10 ng) was used with One Step SYBR^®^ Prime Script^TM^ Kit II (Perfect Real Time) kit according to the manufacturer’s protocol. The realplex 2.2 software (Eppendorf) was used to analyse the data and to calculate cycle threshold (C_t_) values. *Actin1* was taken as housekeeping gene. The change in gene expression was calculated as relative fold change by the comparative C_t_ method^86^.

### Measurement of UPR

β-Galactosidase assay was performed in yeast cell lysates^63^ using 4-methylumbelliferyl β-D-galactopyranoside as the substrate. Specific activity was normalized to protein content.

### Difference gel electrophoresis (DIGE) labelling and 2D gel electrophoresis

Lysis of yeast cells was carried out as described above using 2D gel lysis buffer, pH 8.5 containing urea (7 M), thiourea (2 M), Tris (30 mM) and CHAPS (4%). Contaminants were removed by precipitation using Ready prep 2D-clean up kit (GE Healthcare), following the manufacturer’s protocol. The pellet obtained was resuspended in 2D lysis buffer. Protein content was estimated by Bradford method. Equal amounts of each protein preparation were taken and labelling was carried out with DIGE minimal dye fluors for 30 min on ice in dark using 400 pmol/50 μg protein with different combinations of samples (Cy3 and Cy5 for each set of samples). An internal standard containing equal amount of each sample was labelled with Cy2 in the same manner. The labelling reaction was stopped by adding 1 μl of 10 mM lysine. For each set, all three samples (internal standard Cy2, sample 1 Cy3 and sample 2 Cy5) were mixed and the volume was adjusted using equal amount of 2X sample buffer [urea (7 M), thiourea (2 M), CHAPS (2%), DTT (2%), IPG buffer (2%)] and rehydration buffer. The sample was allowed to rehydrate an 11 cm long Immobiline Dry Strip of pH range 4–7 overnight in Protean IEF (iso-electric focussing) cell chamber (Bio-Rad Laboratories (India) Pvt. Ltd.) set at 20 °C in dark. IEF was performed in dark with some modifications in running program used for yeast proteins with Protean IEF cell. The following running program was selected for IEF run: 500 V, 2 h, slow; 8000 V, 2.5 h, linear; 8000 V-40000 V, 1 h, rapid; 500 V for holding, 5 h to prevent diffusion. Post IEF, strips for each sample set were equilibrated using Tris (75 mM), urea (6 M), glycerol (30%), SDS (2%) with DTT (0.5%), pH 8.8 (Equilibration buffer I, as reducing agent) followed by the same buffer without DTT and with iodoacetamide (1%) (Equilibration buffer II, as alkylating agent), respectively, for 15 min each. Second dimension SDS-PAGE gel (12%) was run using SE600 Ruby (GE Healthcare). The resultant analytical gels were scanned using an image scanner Typhoon Trio (GE Healthcare). The specific excitation and emission wavelengths used were as recommended by the manufacturer. Gel images were scanned at a pixel resolution of 100 microns (μm). Cropped gel images were analyzed using DeCyder 2D software (version 7.2, GE Healthcare).

The differential in-gel analysis (DIA) algorithm detected overlapping spots on a combined image derived from merging individual images from the two samples tagged by Cy3 and Cy5. The biological variation analysis (BVA) algorithm [(DeCyder 2D software, version 7.2) (GE Healthcare)] was used to identify common protein spots across the gels. Various experimental designs were assigned in BVA, allowing the statistical analysis tools to highlight proteins that demonstrated significant difference in expression under different experimental conditions. After scanning, the spots in the gel were visualized by silver staining. Protein spots were excised, digested by trypsin and analyzed by matrix-assisted laser desorption/ionization-time-of-flight (MALDI-TOF/TOF) mass spectrometry [Bruker Daltonics MALDI TOF/TOF UltraflexIII, laser wavelength 337 nm). The peaklist was generated using Bruker Daltonics Flexanalysis; Version 3.3 Build 80 (mass tolerance for precursor ions 50–150 ppm) using the MASCOT search engine. Known contaminants (peaks for α-cyano-4-hydroxycinnimic acid, trypsin and keratin) were excluded. The protein sequence information was used to identify proteins in the *Saccharomyces* Genome database (http://www.yeastgenome.org/).

### Statistical analysis

All values are mean ± standard error of mean (s.e.m.) of at least three independent experiments. Student’s t-test was used to analyze significant difference. A value of p < 0.05 was considered to demonstrate statistical significance.

## Additional Information

**How to cite this article**: Bhadra, A. K. *et al*. Protein aggregation activates erratic stress response in dietary restricted yeast cells. *Sci. Rep.* **6**, 33433; doi: 10.1038/srep33433 (2016).

## Supplementary Material

Supplementary Information

## Figures and Tables

**Table 1:** Differential expression of proteins in yeast cells as indicated.

Spot	Uninduced N v/s DR	Non-DR UI v/s I	DR UI v/s I	Induced N v/s DR
9	2.72↓ in DR	No change	No change	No change
25	2.9↑ in DR	3.54↑ upon induction	No change	1.9↓ in DR
58	No change	15.78↑ upon induction	7.67↑ upon induction	No change
62	15.52↑ in DR	4.26↑ upon induction	2.75↓ upon induction	No change
67	4.39↑ in DR	No change	No change	No change
71	No change	4.85↑ upon induction	2.38↑ upon induction	No change
97	11.75↑ in DR	13.49↑ upon induction	No change	1.5↓ in DR

Fold change and p-values were calculated using BVA algorithm (DeCyder 2D software, version 7.2, GE Healthcare). N: non-dietary restricted, DR: dietary restricted, UI: uninduced, I: induced, ↑ indicates upregulation; ↓ indicates downregulation.

**Table 2:** List of proteins of interest identified by MALDI-TOF/TOF.

Spot	Designation	Systematic name	Protein
9	Atp2	YJR121W	Beta subunit of the F_1_ sector of mitochondrial F_O_F_1_ ATP synthase
25	Eno1/2	YGR254W	Phosphopyruvate hydratase Eno1
58	Arp2	YDL029W	Actin-Related Protein
62	Tpi	YDR050C	Triose phosphate isomerase
67	Utp21	YLR409C	Subunit of U3-containing 90S preribosome and SSU processome complexes
71	Hom6	YJR139C	Homoserine dehydrogenase
97	Fpr1	YNL135C	Peptidyl-prolyl cis-trans isomerase (PPIase)
