# Supplementary material for: Protein aggregation activates erratic stress response in dietary restricted yeast cells
Source: Sci Rep. 2016 Sep 16;6:33433. doi: 10.1038/srep33433 (PMC5025734; doi:10.1038/srep33433)
Supplement: Supplementary Information [file srep33433-s1.pdf]

Supplementary Information

Protein aggregation activates erratic stress response in dietary restricted yeast cells

Ankan Kumar Bhadra, Eshita Das and Ipsita Roy

Table S1. PMF identification.

Spot	Designation	Accession	Matched/Searched	Score	Expect
9	Atp2	gi|398365477	13/39	110	1.3e-06
25	Eno1/2	gi|6321968	7/26	74	0.0045
58	Arp2	gi|6320175	12/26	68	0.00011
62	Tpi	gi|6320255	9/39	99	1.6e-05
67	Utp21	gi|768843067	20/121	66	0.035
71	Hom6	gi|398365575	13/108	83	0.00062
97	Fpr1	gi|6324194	6/57	71	0.011

The mass spectra were internally calibrated using Bradykinin, Angiotensin_II, Angiotensin I, Bombesin, ACTH and Somatostatin. (*m/z 757.39916, 1046.54180, 1296.68480, 1619.82230, 2093.08620, 3147.47100* Da, respectively), resulting in mass errors of less than 18.69 ppm.

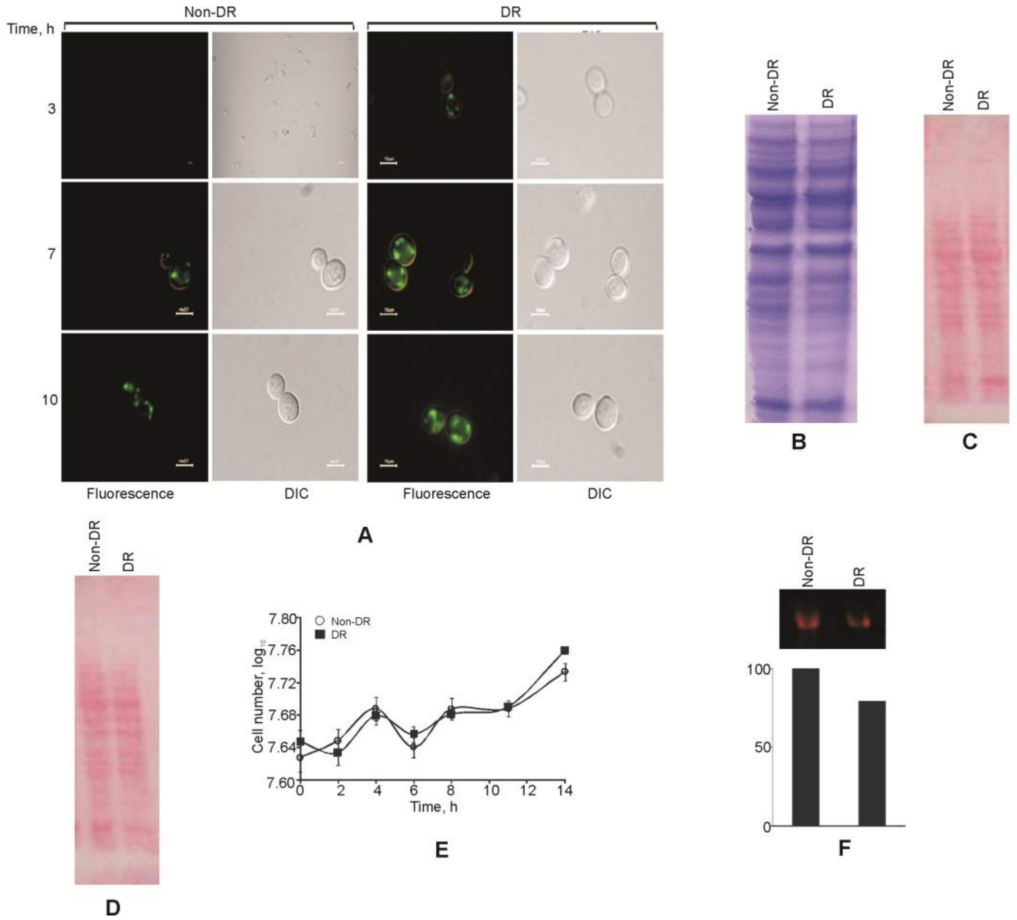

***Fig S1*. Growth and expression of 103Q-htt-EGFP in yeast cells.** (A) Fluorescence micrographs of yeast cells grown under non-DR and DR conditions induced for different time periods were recorded. Images were visualized under 100 X objective and 10 X eyepiece. Bar = 10 μm. (B) Loading control (Coomassie staining) of gel shown in Fig. 1B. (C) Loading control (Ponceau S staining) of membrane shown in Fig. 1C. (D) Loading control (Ponceau S staining) of membrane shown in Fig. 1D. (E) Growth curves of *Saccharomyces cerevisiae* cells. Yeast cells were grown till A_{600} 0.8. Expression of 103Q-htt was induced with SC-URA+2% galactose and cell growth was monitored. Values shown are mean±s.e.m. of three independent experiments. (F) Native PAGE analysis of soluble fraction of yeast cell lysates expressing 103Q-htt-mRFP was carried out. Yeast cells were transformed with pRS315-*103Q-htt-mRFP* (*CUP1* promoter, *LEU2* selection marker) (described in detail in Supplementary Reference 1) and induced with 500 μM Cu^{2+} ions for protein expression for 7 h. Densitometry of bands was performed using ImageQuantTL (GE Healthcare).

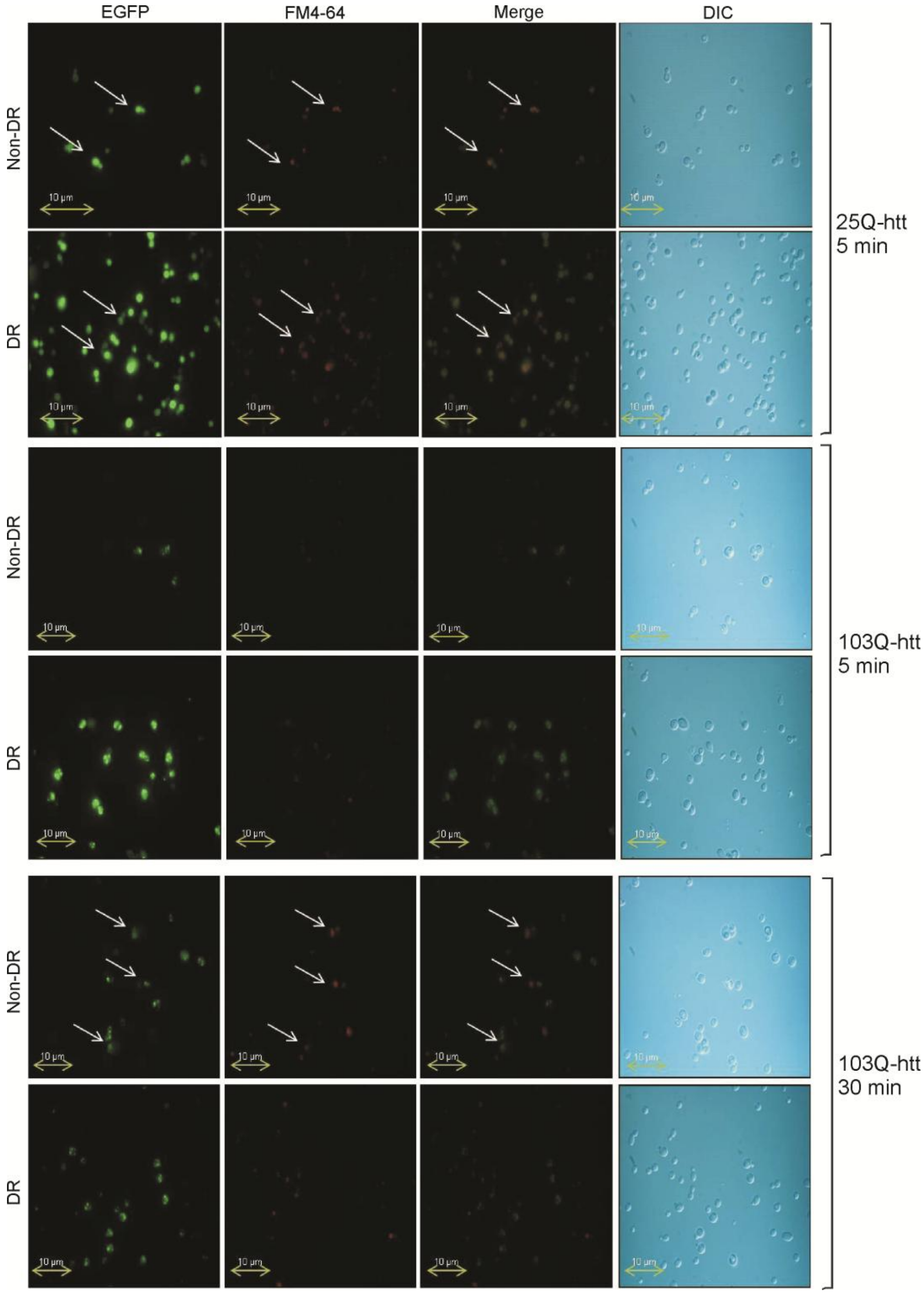

***Fig S2*. Endocytosis was monitored in yeast cells expressing 25Q-htt or 103Q-htt grown under non-DR and DR conditions**. Internalization of FM4-64 was followed for different time periods. Images were visualized under 100X objective and 10X eyepiece. Bar=10 µm. Arrows indicate representative cells expressing 25Q-htt or 103Q-htt where internalization of FM4-64 was observed.

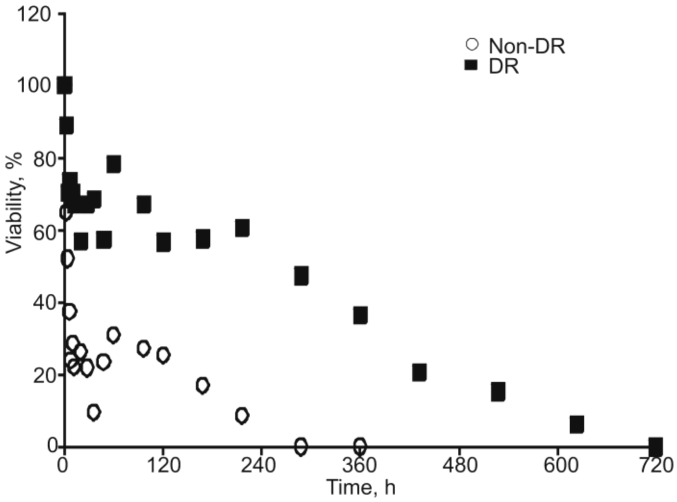

***Fig S3*. Measurement of chronological life span of uninduced yeast cells grown under non-DR and DR conditions**. 100% represents viability of starting cells in each case.

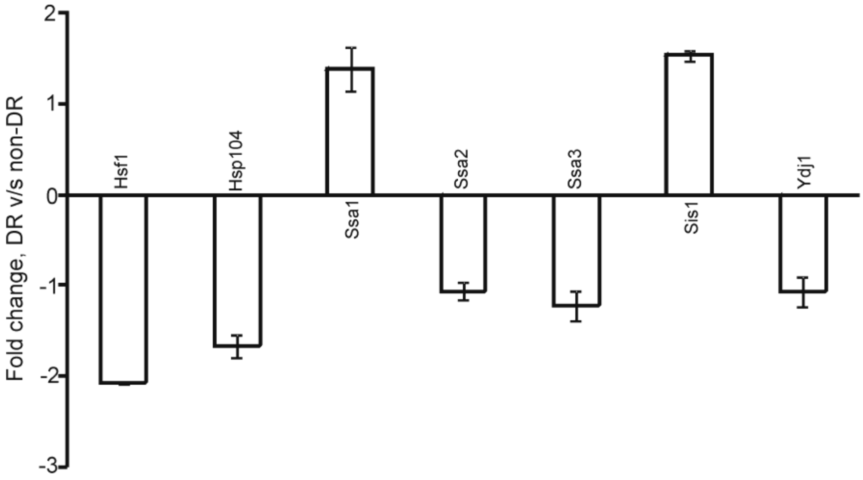

***Fig S4*. Comparative analysis of expression of some genes involved in heat shock response pathway in yeast cells expressing 103Q-htt grown under non-DR and DR conditions.** Differential expression of genes expressed in cells grown under DR condition was measured against that under non-DR condition.

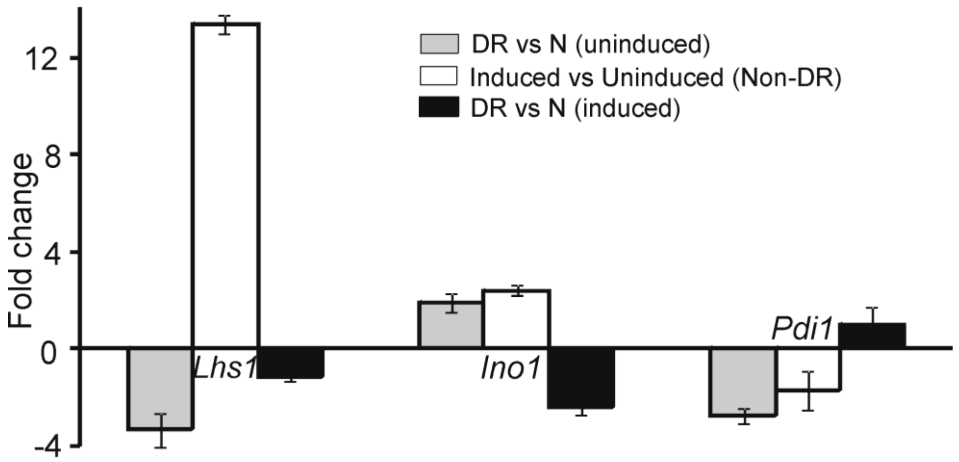

***Fig S5*. Comparative analysis of expression of some genes acting as targets of the unfolded protein response pathway in yeast cells.**

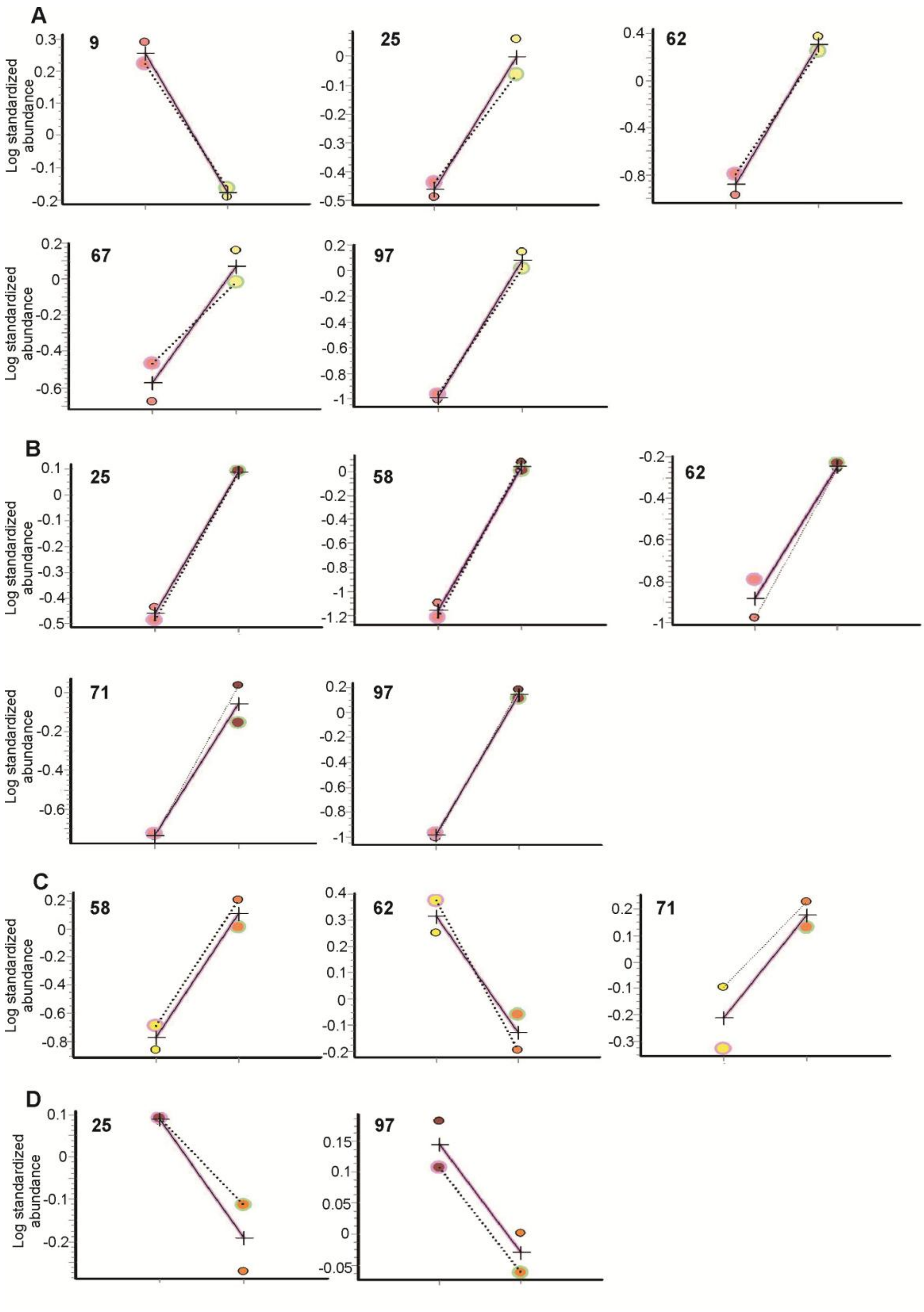

***Fig S6*. Relative protein abundance of differentially expressed spots**. Comparison shown between (A) uninduced yeast cells grown under non-DR and DR conditions, (B) uninduced and induced yeast cells grown under non-DR condition, (C) uninduced and induced yeast cells grown under DR condition, and (D) induced yeast cells grown under non-DR and DR conditions. Spot numbers (orange circles in gel images of

respective conditions, as shown in Fig. 5) are indicated on the figure. Dotted line represents the difference in expression between individual samples. Solid line indicates the mean difference in expression between two different conditions/samples under analysis.

Supplementary Reference

1. Saleh, A.A., Bhadra, A.K. & Roy, I. Cytotoxicity of mutant huntingtin fragment in yeast can be modulated by the expression level of wild type huntingtin fragment. *ACS Chem. Neurosci.* **5**, 205-215 (2014).
